# Effect of different ventilation modalities on the early prognosis of patients with sleep apnea after acute ischemic stroke–––protocol for a prospective, open-label and randomised controlled trial

**DOI:** 10.1186/s12883-023-03117-6

**Published:** 2023-06-06

**Authors:** Zhuanyun Li, Ming Pang, Yaling Yu, Tianfeng Peng, Zhenghao Hu, Ruijie Niu, Xiaorong Wang, Jinnong Zhang

**Affiliations:** 1 Department of Emergency Medicine, Union Hospital, Tongji Medical College, Huazhong University of Science and Technology, Wuhan, China; 2 National Clinical Research Center for Geriatrics, West China Hospital, Sichuan University, Chengdu, China; 3 Department of Respiratory and Critical Care Medicine, Union Hospital, Tongji Medical College, Huazhong University of Science and Technology, Wuhan, China

**Keywords:** Sleep apnea, Acute ischemic stroke, High-flow nasal cannula, Nasal continuous positive airway pressure, Randomised controlled study

## Abstract

**Background:**

Sleep apnea is highly prevalent after acute ischemic stroke (AIS) and has increased stroke-related mortality and morbidity. The conventional sleep apnea treatment is continuous positive airway pressure (CPAP) ventilation. However, it is poorly tolerated by patients and is not used in all stroke patients. This protocol describes the impact of high-flow nasal cannula (HFNC) oxygen therapy compared to nasal continuous positive airway pressure (nCPAP) ventilation or usual care on the early prognosis of patients with sleep apnea after AIS.

**Methods:**

This randomised controlled study will be conducted in the intensive care unit of the Department of Neurology at the Wuhan Union Hospital. According to the study plan, 150 patients with sleep apnea after AIS will be recruited. All patients are randomly allocated in a 1:1:1 ratio to one of three groups: the nasal catheter group (standard oxygen group), the HFNC group, and the nCPAP group. Patients receive different types of ventilation after admission to the group, and their tolerance while using the different ventilation is recorded. Patients will be followed up by telephone three months after discharge, and stroke recovery is recorded. The primary outcomes were 28-day mortality, the incidence of pulmonary infection and endotracheal intubation.

**Discussion:**

This study analyses different ventilation modalities for early interventions in patients with sleep apnea after AIS. We will investigate whether nCPAP and HFNC reduce early mortality and endotracheal intubation rates and improve distant neurological recovery in patients.

**Trial registration:**

This trial was registered at ClinicalTrials.gov (NCT05323266; 25 March 2022).

## Background

Sleep apnea is one of the most common chronic diseases, affecting approximately one billion people worldwide [1]. However, its social and economic consequences mean that sleep apnea is a significant public health problem [2]. Sleep apnea, particularly obstructive sleep apnea (OSA), is more common in patients with acute ischemic stroke (AIS) and transient ischemic attack (TIA) (60–80%) [3, 4] and is associated with an increased risk of poor recovery, stroke recurrence and death [5, 6]. Currently, the oxygen therapy for patients with sleep apnea after AIS is continuous positive airway pressure (CPAP) [7, 8].

Early use of CPAP in patients with first ischemic stroke combined with sleep apnea (apnea–hypopnea index (AHI) ≥ 20 events/h) has been shown to improve neurological function significantly. In addition, CPAP prolongs the mean time from stroke onset to the first cardiovascular event [9]. However, CPAP could be unsuitable for patients with sleep apnea, as the mask and the elevated airway pressure during CPAP may induce sleep disturbances [10]. High-flow nasal cannula (HFNC) therapy is a new non-invasive respiratory support option that reduces upper airway collapse and lowers carbon dioxide levels at low positive pressure levels [11]. HFNC effectively reduces the AHI in children with sleep apnea, consistent with CPAP in children aged 7–14 [12, 13].

Therefore, we propose to conduct this clinical study in the intensive care unit of the Department of Neurology, Wuhan Union Hospital. The early prognostic value of different ventilation modalities on patients with sleep apnea after AIS will be clarified, laying the foundation for guiding clinical practice later.

## Methods and design

### Study design and setting

**Fig. 1:**
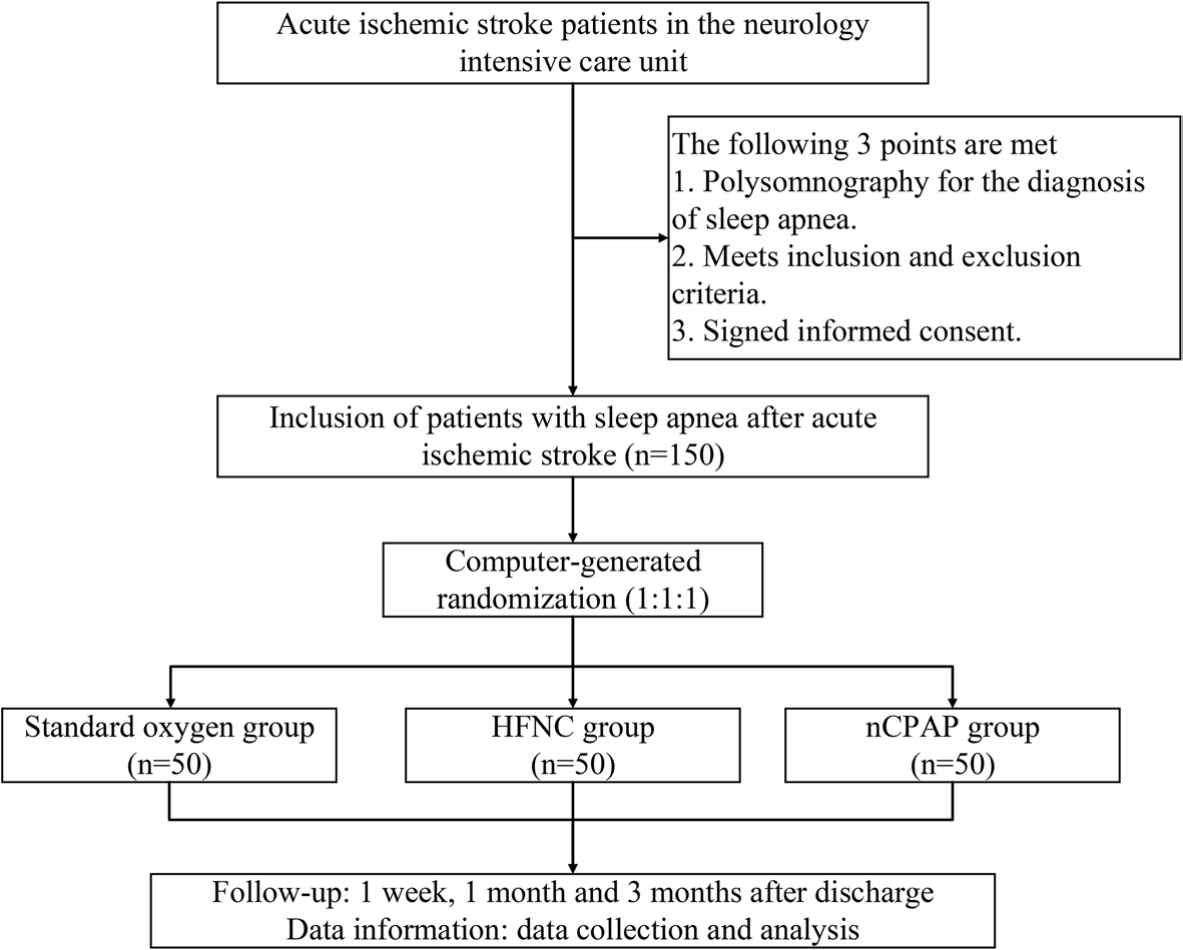
Flow chart of the clinical trial

This prospective, open-label and randomised controlled trial will be conducted in the intensive care unit of the Department of Neurology at Wuhan Union Hospital. Enrolment of all participants begins on May 1, 2022. All participants with sleep apnea included are randomised according to 1:1:1 into the standard oxygen therapy group, the HFNC group and the nasal continuous positive airway pressure (nCPAP) group. Figure 1 demonstrates the flow chart of the study.

### Recruitment

In the clinical trial, we recruit participants with sleep apnea after AIS in the intensive care unit of the Department of Neurology, Wuhan Union Hospital. Recruitment is planned from May 1, 2022, to April 31, 2023. The recruited participants will be randomly grouped according to 1:1:1 into the standard oxygen therapy group, the HFNC group, and the nCPAP group. All participants will be informed in detail about the clinical trial and will provide written informed consent before recruitment. All patients will be followed up for three months after discharge, and the end date of post-discharge follow-up will be 31 July 2023. A detailed description of the trial's registration, interventions and assessment schedule is given in Table 1.

**Table 1:** Timeline of the study

	**STUDY PERIOD**						
	**Enrolment**	**Allocation**	**Post-allocation**				**Close-out**
**TIMEPOINT**	***May 2022—April 2023***	**May 2022—April 2023**	***t***_***1***_	***t***_***2***_	***t***_***3***_	***t***_***4***_	***August 2023***
**ENROLMENT:**							
**Recruitment**	X						
**Eligibility screen**	X						
**Informed consent**	X						
**Allocation**		X					
**INTERVENTIONS:**							
**standard oxygen group**			X	X	X	X	
**HFNC group**			X	X	X	X	
**nCPAP group**			X	X	X	X	
**ASSESSMENTS:**							
**Baseline questionnaire**	X						
**Follow-up questionnaire**			X	X	X	X	
**Analyse data**							X

*t*_*1*_: treatment in hospital

*t*_*2*_: 1 week after discharge

*t*_*3*_: 1 month after discharge

*t*_*4*_: 3 months after discharge

### Inclusion criteria

1. Age ≥ 18 years; 2. Clinical diagnosis of AIS; 3. Diagnosis of AIS confirmed by CT and MRI; 4. National Institutes of Health Stroke Score (NIHSS) baseline score of 2–20; 5. State of consciousness (Glasgow Coma Score (GCS) of ≥ 9); 6. Semi-quantitative cough strength score of ≥ 2.

### Exclusion criteria

1. Pre-existing obstructive sleep apnea; 2. Suspected sleep disorders other than sleep apnea (e.g., episodic sleeping sickness); 3. Respiratory distress requiring mechanical ventilation; 4. Oxygen-dependent chronic obstructive pulmonary disease; 5. Pregnancy; 6. Intracranial haemorrhage; 7. Inability to use a nasal mask or mask (e.g., facial trauma); 8. Patients who died within 24 h of admission; 9. History of other neurological disorders such as Parkinson's, neuro infection, and neuromuscular disease patients; 10. Hospice care or comfort measures only; 11. Inability to provide informed consent; 12. Inability to provide valid information; 13. Suicidal ideation.

### Withdraw criteria

1. Unable to tolerate the clinical trial and requesting an early withdrawal. 2. Patient is unsuitable for further trials due to deterioration. 3. Poor compliance with the follow-up plan.

### Sample size

The sample size is calculated using the Contingency Table (Chi-Square Tests) in the PASS 15.0 software. A literature review showed that nCPAP was approximately 65% effective for neurological recovery in stroke patients and 55% effective for HFNC, compared to 40% in the standard oxygen therapy group [14, 15]. In this study, the test level α = 0.05 and the test efficacy 1-β = 0.9. The final sample size is expected to be 150 cases, considering a 10% loss to follow-up rate, including the possibility of deterioration after admission and loss to follow-up after discharge.

### Randomisation and blinding

All participants are informed of the trial's purpose and sign an informed consent form. Participants will be randomised by block randomisation in the randomisation tool (www.randomisation.com). The randomisation sequence is assigned by an independent statistician using a random block size of 6 and 9 in a 1:1:1 fashion. The randomised numbers are placed in opaque sealed envelopes, and participants entering the group will be randomly given one of the envelopes and a grouping result based on the number. The data analysts in this study will not be involved in any interventions and will remain blinded to group allocation. The trial is open, as the researcher and participants cannot mask group allocation.

### Control group

In the trial, participants in the control group are treated with either conventional nasal cannula oxygen therapy or mask oxygen therapy. Treatment and care remained the same, except that the oxygen therapy is administered differently from the intervention groups. Participants will be monitored during their stay in the hospital and withdrawn from the clinical trial if necessary.

### Intervention groups

Participants in the intervention groups will be randomised to HFNC oxygen therapy and nCPAP oxygen therapy. In participants treated with HFNC oxygen therapy, the initial flow rate is set at 20L/min. Titration is performed during sleep, with each flow rate increasing by 10L/min until 60L/min, attempting to eliminate apnea, hypopnea, and hypoventilation in the supine position or during rapid eye sleep. When participants are treated with nCPAP, initial pressure support of 6-8cmH_2_O is first provided via a nasal mask. The nCPAP pressure is titrated and adjusted during sleep until apnea, hypopnea, and hypoventilation are eliminated. During titration, participants were sleep monitored using full-night polysomnography, and the number of apnea episodes, hypopnoea and hypoventilation episodes were recorded. When participants have completed titration, the setting of the optimal oxygen therapy mode parameters is recorded. Participants will be continuously ventilated according to the optimal oxygen therapy mode at the time of titration during their hospital stay. Participants will be monitored for changes in condition and oxygen saturation during the implementation of both interventions, and clinical trials will be terminated when necessary.

### Study outcomes

#### Primary outcomes

1. 28-day mortality rate; 2. Tracheal intubation rate (Rate of tracheal intubation in each group after one week using different ventilation methods); 3. Pulmonary infection rate (Rate of pulmonary infections in each group after one week using different ventilation methods).

#### Secondary outcomes

1. National Institute of Health stroke scale (NIHSS). The NIHSS is used to evaluate the severity of stroke patients. Changes in NIHSS scores after one week in acute ischemic stroke patients with different ventilation modes. NIHSS scores range from 0–42, with higher scores indicating more severe neurological damage. 2. Barthel index. The Barthel index is used to assess the ability of stroke patients to live independently. This study assesses whether the quality of life of patients with acute ischemic stroke improves at one month and three months. 3. Sleep apnea symptoms. To assess changes in sleep apnea symptoms in patients with acute ischemic stroke. After one week of treatment with different ventilation modalities, polysomnography will be performed again to assess whether sleep has improved in stroke patients by calculating the magnitude of the AHI value. The minimum value of the AHI is 0, and there is no maximum value. A higher AHI value indicates more severe sleep apnea symptoms; 4. Time of hospitalization; 5. Cost of hospitalization; 6. Neurological recovery (mRS).

### Quality control

Differences in scores may result from the clinical experience of the doctors. Therefore, each patient's assessment is administered by the same doctor to ensure accuracy and consistency of scoring each time. Additionally, the performer is trained several times prior to conducting the assessment.

### Data collection and follow-up

All participants in the clinical trial are assessed at baseline, and a specialist team member records information. Trial data includes baseline information (age, sex, height, weight, heart rate, blood pressure), previous medical history (history of surgery, family history, history of smoking and alcohol consumption), laboratory findings, imaging findings (brain MRI or CT), polysomnography findings, NIHSS score, mRS, Barthel Index, acute physiology and chronic health evaluation (APACHE) II score, GCS score, etc. Telephone follow-up is conducted one week, one month and three months after the participants are discharged from the hospital according to the plan developed for the study to assess the participants' neurological recovery and prognosis.

### Data management

All data from the clinical study will be entered directly into the computer via EpiData V.3.1 (EpiData Association, Odense, Denmark). The study team will enter the data, and the data's accuracy will be verified by the study's principal investigator (PI). All data is protected and stored securely at the study site. Researchers can only access information by entering the correct username and password. The participants' privacy is fully protected in this study, and all participants will not be identified by name. Post-discharge follow-up is achieved through each participant's unique number and telephone number at the time of admission. The PI of the study will have access to the final study data set.

### Data and safety monitoring

An independent data monitoring committee includes neurologists, respiratory and critical care physicians, emergency medicine physicians, statisticians and data management staff. The committee will meet twice during the year, and its main task is to monitor and review the data.

### Statistical analysis

The final data analysis will be done by statisticians uninvolved in the clinical trial and kept blind. The three data sets will be done by one-way ANOVA (normally distributed data) or Kruskal–Wallis statistics (non-parametric data). Categorical variables are done via chi-square tests. In addition, logistic regression and propensity score matching (PSM) will be used to adjust for the effect of multivariate risk factors on early clinical outcomes. Survival analysis will be performed using the Kaplan–Meier method. All statistical analyses will be performed by SPSS 22.0 (IBM SPSS Statistics 22.0, SPSS Inc., Chicago, IL) and R language (version 4.1.3, www.R-project.org/). Statistical significance is expressed as *p* < 0.05.

### Missing data plan

During the clinical trial, if data are missing, we will record the reason for the absence and the amount of data. During follow-up, if < 10% of participants are missing, we will use the complete case method. If ≥ 10% of participants are missing, and we will use the multiple imputation method to impute missing values.

### Adverse events

During the trial, a senior practitioner will assess any adverse events experienced by participants, and the participant's family will be informed promptly. Participants will be withdrawn from the trial in a timely manner if their condition worsens or they are unfit to continue participating.

### Modification of the protocol

Any modification to the protocol that may affect the conduct of the study, the potential benefit to patients or may affect patient safety needs to be approved by the Ethics Committee before implementation.

## Discussion

Our research team will use this clinical trial to illustrate the impact of different ventilation modalities on the early prognosis of patients with post-stroke sleep apnea and to bring clinical benefit to a broader range of patients. In this clinical trial, we will primarily use nCPAP and HFNC interventions in stroke patients. The early use of nCPAP and HFNC in stroke patients was assessed by comparing the outcomes of 28-day mortality, tracheal intubation rate, pulmonary infection rate and neurological recovery in patients with sleep apnea after AIS.

Currently, sleep apnea after AIS is a common clinical condition, and CPAP therapy is a more effective treatment [16]. A meta-analysis (10 randomised controlled trials [RCTs], *n* = 564 stroke patients) showed that CPAP therapy reduced stroke severity and improved neurological function in patients with post-stroke sleep apnea [17]. However, some patients had poor compliance due to excessive airflow pressure during CPAP therapy and could not use it for prolonged periods. Therefore, HFNC may be the best option for patients who cannot tolerate CPAP therapy.

Due to its simplicity, broad applicability to the population and good compliance at different levels, HFNC has been widely used in clinical practice. One study found that HFNC was not inferior to CPAP in neonates who were extubated after mechanical ventilation [18, 19]. HFNC is also better tolerated and more comfortable in COPD patients with severe hypercapnia and respiratory failure [20]. However, whether HFNC is clinically useful in patients with post-stroke sleep apnea and whether they have the same therapeutic benefit as CPAP.

In summary, the results of our clinical trial will clarify the value of the early use of nCPAP and HFNC in patients with sleep apnea after AIS, providing more options for ventilation in patients with stroke and improving their early prognosis.

## Data Availability

This clinical trial is ongoing. The dataset or analysis of the results of this trial upon completion is available from the corresponding author by reasonable request.

## Abbreviations

AIS: Acute ischemic stroke
CPAP: Continuous positive airway pressure
HFNC: High-flow nasal cannula
nCPAP: Nasal continuous positive airway pressure
OSA: Obstructive sleep apnea
TIA: Transient ischemic attack
AHI: Apnea–hypopnea index
NIHSS: National institutes of health stroke score
GCS: Glasgow coma score
mRS: Modified Rankin scale
APACHE II score: Acute physiology and chronic health evaluation II score
PSM: Propensity score matching
